# Association Between Survival After Living Donor Liver Transplantation and Recipient Systemic Inflammation and Body Composition

**DOI:** 10.3390/jcm14165889

**Published:** 2025-08-20

**Authors:** Jae Hwan Kim, Yeon Ju Kim, Hye-Mee Kwon, Kyung-Won Kim, Jin YanZhen, Sa-Jin Kang, In-Gu Jun, Jun-Gol Song, Gyu-Sam Hwang

**Affiliations:** 1 Department of Anesthesiology and Pain Medicine, Inje University Haeundae Paik Hospital, Busan 48108, Republic of Korea; 2 Laboratory for Cardiovascular Dynamics, Department of Anesthesiology and Pain Medicine, Asan Medical Center, University of Ulsan College of Medicine, 88 Olympic-ro 43-gil, Seoul 05505, Republic of Korea; yjans@amc.seoul.kr (Y.J.K.);; 3 Asan Image Metrics, Department of Radiology, Asan Medical Center, University of Ulsan College of Medicine, Seoul 05505, Republic of Korea; 4 Department of Anesthesiology and Pain Medicine, Chung-Ang University Gwangmyeong Hospital, Gwangmyeong-si 14353, Republic of Korea

**Keywords:** liver transplantation, recipient, sarcopenia, systemic inflammation, neutrophil-to-lymphocyte ratio, correlation, mortality

## Abstract

**Background/Objectives:** Preoperative sarcopenia in liver transplantation (LT) recipients is an important prognostic factor of LT outcomes. Systemic inflammatory status (SIS) has been proposed as a unifying mechanism for skeletal muscle loss; thus, considering SIS and sarcopenia together may enhance prognosis assessment in patients undergoing LT. Herein, we aimed to describe the relationship between the SIS and skeletal muscle index (SMI) with short-term and long-term mortality post-living donor LT (LDLT). **Methods:** In total, 3387 consecutive adult LDLT recipients were retrospectively evaluated. The neutrophil-to-lymphocyte ratio (NLR, using a cut-off of 3) was utilized as an SIS. SMI was calculated using computed tomography scans, measured at the third lumbar vertebra; sex-specific cut-offs were determined from contemporary donors. Univariate and multivariable Cox proportional hazard analyses were performed. **Results:** Decreasing SMI was associated with increasing NLR. Increasing NLR and decreasing SMI both showed dose-dependent relationships with a risk of 90-day mortality. Within sarcopenic patients, NLR > 3 (vs. NLR ≤ 3) was associated with higher 90-day (9.3% vs. 3.5%, *p* = 0.049) and overall mortality (28.4% vs. 19.1%, *p* = 0.045). Sarcopenia and NLR > 3 (vs. neither) were independent predictors of 90-day mortality (hazard ratio [HR] 2.48 [1.40–4.40], *p* = 0.002) and overall mortality (HR, 1.81 [1.37–2.38], *p* < 0.001) after multivariable adjustment. When stratified by age, sex, and MELD score, the association between sarcopenia and overall mortality persisted in all subgroups, with the highest risk observed in women (HR 3.43, 95% CI 1.83–6.43). **Conclusions:** Sarcopenia, with the systemic inflammatory response, nearly doubled the risk of 90-day and overall mortality post-LT, proposing that these readily available biomarkers are a practical index for predicting survival post-LT. Considering that these are potentially modifiable factors, our result may provide a new therapeutic target to improve survival post-LT.

## 1. Introduction

Liver transplantation (LT) greatly increases the survival of patients with end-stage liver disease (ESLD) and has been established as the gold standard treatment. However, the identification of patients at a high risk of adverse outcomes and mortality after LT is a clinical priority. Currently, the Model for End-stage Liver Disease (MELD) score is a widely used prognostic score to evaluate cirrhosis severity. However, the MELD score, which considers creatinine, bilirubin, and the internationalized normalized ratio, lacks the evaluation of the nutritional status and may thus not fully reflect patients’ general condition [1,2].

Skeletal muscle mass has emerged as a proxy for patient fragility. Muscle mass has been quantified using measurements at the third lumbar vertebra level from computed tomography (CT) scans, a routine diagnostic imaging modality in patients with ESLD [3]. The skeletal muscle index (SMI) is widely used to assess sarcopenia in patients undergoing liver transplantation [4]. While body mass index (BMI) is another commonly used metric of body composition, it does not accurately reflect muscle quantity or quality. As such, patients with normal or elevated BMI may still have significant muscle depletion—a condition known as sarcopenic obesity. An accumulating body of evidence supports the presence of fragility (i.e., reduced muscle mass), which is not captured by the MELD score, to be an independent prognostic factor of patients with liver cirrhosis and survival after LT [4,5,6].

Similarly, the neutrophil-to-lymphocyte ratio (NLR) is suggested to be a biomarker of systemic inflammatory status. In advanced liver cirrhosis (LC), immune dysregulation is frequent and serves as a pathogenetic contributor regardless of the presence of overt infection [7,8] and is related with poor prognosis [9,10]. Escalated NLR has a parallel relationship with an escalated degree of immune dysfunction [11] and has been validated as a significant prognostic factor in LC prognosis [11,12,13,14] and survival after LT [15].

Currently, it is speculated that the systemic inflammatory status both triggers and is progressed by muscle degradation. The systemic inflammatory response is recognized as a unifying mechanism for weight loss and muscle breakdown. Specifically, pro-inflammatory cytokines and growth factors released as part of the systemic inflammatory response have considerable catabolic effects on host metabolism. Simultaneously, low muscularity could contribute to local inflammation in the muscle, worsening muscle degradation, triggering systemic inflammation [16], and eventually causing a vicious cycle leading to impaired prognosis [17].

Therefore, in this study, we aimed to investigate the independent and combined relationships between the systemic inflammatory status and skeletal muscle index (SMI) with short-term and long-term mortality after living donor LT (LDLT). Furthermore, sensitivity analyses with subgroups of age, sex, and severity of liver cirrhosis (MELD score) were performed.

## 2. Materials and Methods

### 2.1. Patients

Data of 3940 LDLT recipients and corresponding donors between January 2008 and January 2018 were collected using a computerized patient data-recording system (Asan Biomedical Research Program, Seoul, Republic of Korea). The exclusion criteria were as follows: age ≤ 18 years; signs of preoperative overt infection, such as pneumonia and spontaneous bacterial peritonitis, owing to the risk of falsely elevated NLR; and incomplete laboratory data. Finally, 3387 LDLT recipients and 3387 corresponding donors were enrolled. This study was approved by the Institutional Review Board of Asan Medical Center, Seoul, Republic of Korea (#2018-1108), and the requirement for written informed consent was waived owing to the retrospective nature of this study. This study was conducted in accordance with the ethical standards of the Helsinki Declaration of 1975.

### 2.2. Sarcopenia Definition and Assessment

Muscle mass was assessed from a CT scan. Among CT scans obtained before LT, those closest to the surgery date were used. An experienced image analyst (You-Sun Ko) selected an axial CT slice at the third lumbar vertebra (L3) inferior endplate level and analyzed the all-muscle area (cm^2^) according to a predetermined threshold (−29 to 150 Hounsfield units) using artificial intelligence software (AID-UTM, iAID Inc., Seoul, Republic of Korea). The analyzed result was defined as the skeletal muscle area (SMA); SMA (cm^2^) was adjusted to the height (m^2^), and the results were defined as the SMI (cm^2^/m^2^).

If there were difficulties in determining the L3 level because of vertebral–anatomy variation, an experienced radiologist (Kyoung-Won Kim) identified the exact L3 level. Our cut-off value for sarcopenia was determined to be 2 standard deviations from the mean baseline value according to recommendations from the European Working Group on Sarcopenia in Older People [18].

### 2.3. Systemic Inflammatory Marker

The systemic inflammatory marker was the NLR derived from laboratory values obtained from routine blood test results collected 1 day before surgery. The neutrophil count was divided by the lymphocyte count to calculate the NLR, and patients were grouped as high NLR (>3) or low NLR (≤3), which has been previously reported as the “normal” cut-off value [19].

### 2.4. Data Collection

Data on patient demographics and perioperative variables were collected using the electronic medical records of our institution. Patient characteristics included age, sex, body mass index (BMI), diabetes mellitus (DM), hypertension (HTN), MELD score, beta-blocker use, diuretic use, and cirrhosis etiology. Preoperative variables included serum hemoglobin, albumin, and sodium levels. Donor-related variables included age, sex, BMI, graft to recipient weight ratio, and donated liver type. Intraoperative variables included operation time, cold ischemic time, warm ischemic time, postreperfusion syndrome, and massive transfusion. Massive transfusion was defined as intraoperative transfusion of > 10 units of packed red blood cells.

### 2.5. Outcome Measures

The primary outcome was 90-day mortality and overall mortality following LT. Patients were followed up until 28 February 2020.

### 2.6. Statistical Analysis

Continuous variables were described as the mean ± standard deviation (SD) or median and interquartile range (IQR) according to their normality, and categorical variables were described as frequencies (%). The Student’s *t*-test, Mann–Whitney *U*-test, chi-square test or Fisher exact test were used for between-group comparisons, as appropriate.

To compare survival, we classified an NLR of 3 or greater and sarcopenia in 3 categories (both, either sarcopenia or inflammatory state only, neither). Univariate and multivariate Cox regression analyses were performed to estimate the association with 90-day mortality and overall mortality. Kaplan–Meier curves were used to compare the cumulative 90-day mortality and overall mortality according to the above mentioned categories. The log-rank test was used to identify statistical significance.

All reported *p* values of < 0.05 were considered statistically significant. Data manipulation and analyses were performed using R software (version 3.6; R Foundation for Statistical Computing, Vienna, Austria)

## 3. Results

### 3.1. Demographics

In total, 3569 patients underwent LT between January 2004 and December 2023 and were enrolled. The exclusion criteria were age ≤ 18 years (*n* = 209); signs of preoperative overt infection, such as pneumonia and spontaneous bacterial peritonitis (*n* = 107), owing to the risk of falsely elevated NLR; and incomplete laboratory data (*n* = 7). Finally, 3387 patients were included in the final analysis.

Table 1 presents the demographic and perioperative characteristics of patients according to sarcopenia and NLR categories. In patients without sarcopenia, high NLR was associated with significantly lower BMI (*p* < 0.001), higher MELD scores (*p* < 0.001), and a higher prevalence of diabetes (*p* = 0.008). These patients showed a higher frequency of alcohol-related cirrhosis (*p* < 0.001) and a lower prevalence of HBV infection (*p* < 0.001) and had lower hemoglobin (*p* < 0.001), lower sodium levels (*p* < 0.001), more deceased-donor grafts (*p* < 0.001), greater transfusion requirements (*p* < 0.001), and more frequent postreperfusion syndrome (*p* = 0.001). Of patients with sarcopenia, those with higher NLR had higher MELD scores (*p* < 0.001), lower hemoglobin (*p* = 0.002), and lower sodium levels (*p* = 0.003). High NLR was also associated with a higher likelihood of alcohol-related liver disease (*p* = 0.047), deceased-donor grafts (*p* < 0.001), and need for massive transfusion (*p* < 0.001).

### 3.2. Mortality Compared According to Prevalence of Sarcopenia and NLR Categories (≤3, >3)

**Figure 1:**
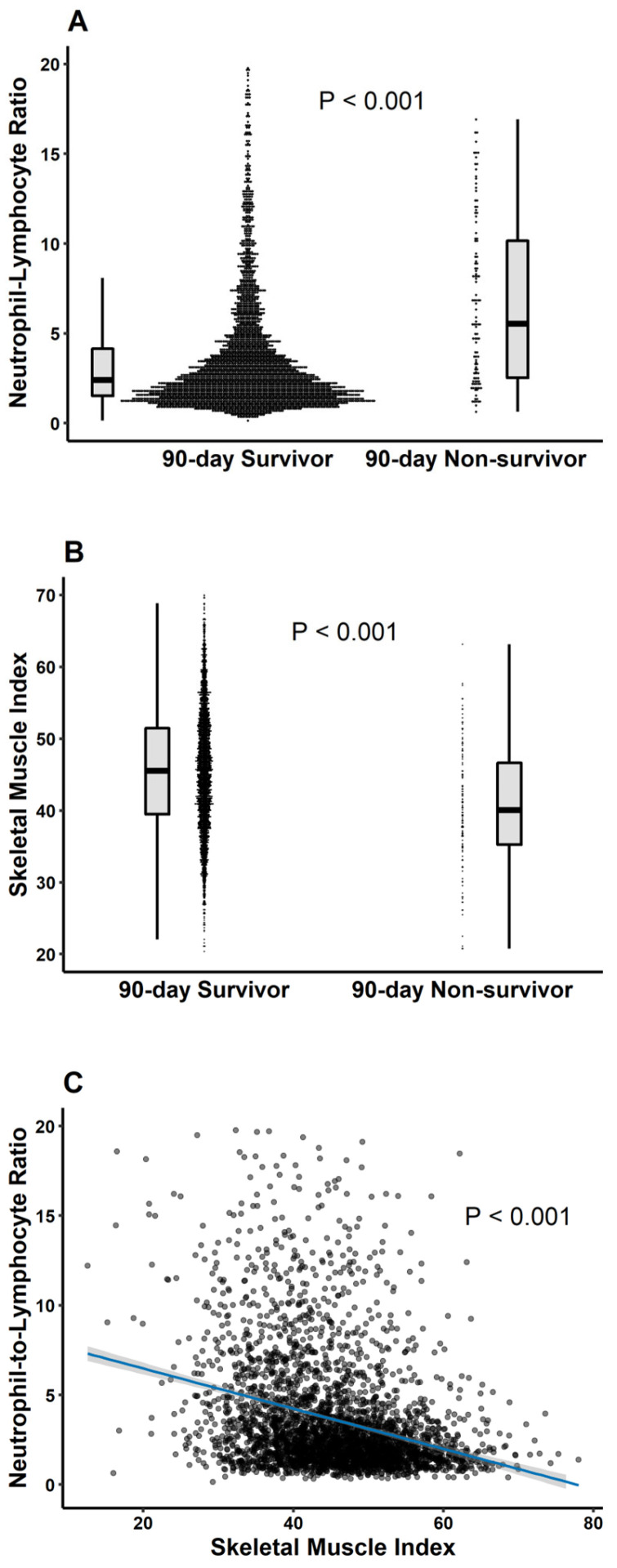
Comparison of (**A**) NLR and (**B**) skeletal muscle index between 90-day survivors and non-survivor. (**C**) Correlation between NLR and skeletal muscle index using scatter line graph.

Importantly, among patients without sarcopenia, both 90-day mortality (5.6% vs. 1.6%, *p* < 0.001) and overall mortality (17.6% vs. 12.9%, *p* = 0.001) were significantly higher in those with high NLR. Mortality outcomes were consistently worse in patients with sarcopenia: 90-day mortality was 9.3% in the high-NLR group versus 3.5% in the low-NLR group (*p* = 0.049); overall mortality was 28.4% vs. 19.1% (*p* = 0.045), respectively. Comparisons between 90-day survivors and non-survivors demonstrated significant differences in both NLR and SMI. Survivors at 90 days showed significantly lower NLR (Figure 1A) and higher SMI (Figure 1B) compared to non-survivors (*p* < 0.001 for both). Additionally, a negative correlation was observed between NLR and SMI (Figure 1C), supporting the inverse relationship between systemic inflammation and muscle mass.

**Figure 2:**
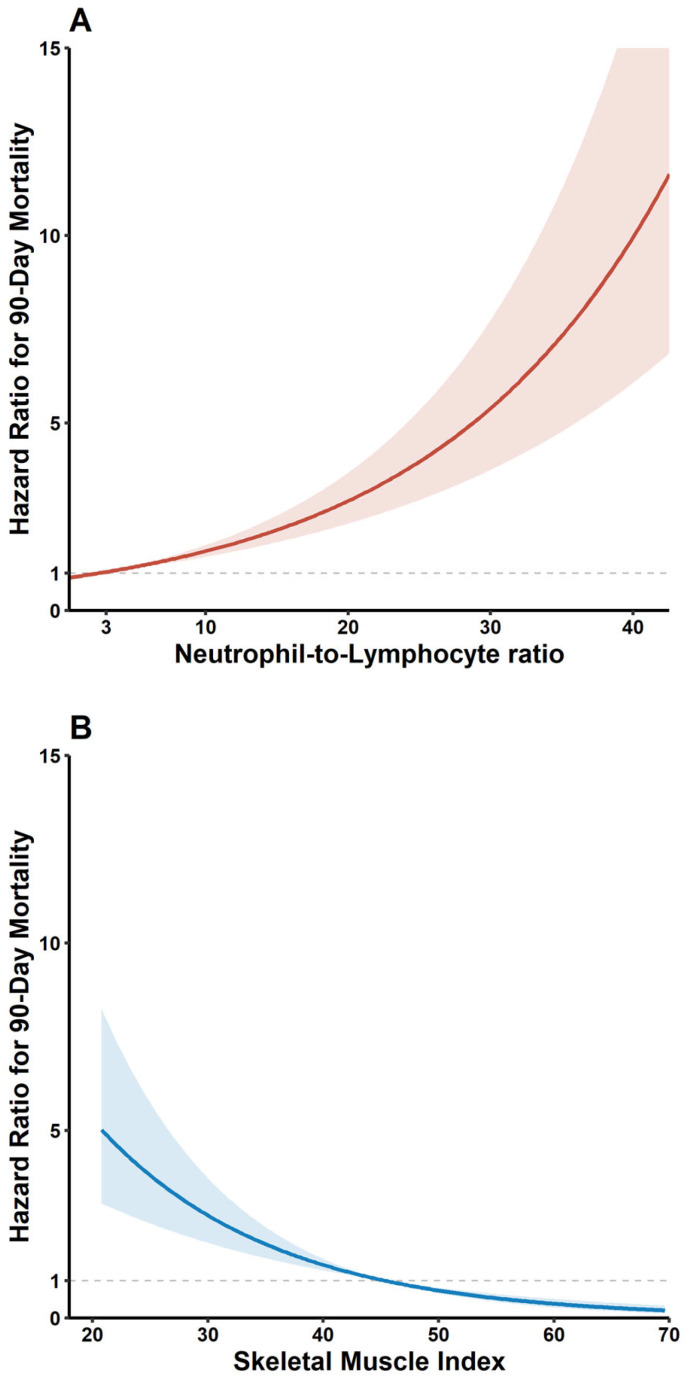
Multivariate-adjusted hazard ratio plot showing the relationship between 90-day mortality and (**A**) NLR, and (**B**) skeletal muscle index.

The red and blue lines in Figure 2 represent the spline curves for NLR (A) and SMI (B), respectively, with the shaded areas indicating the 95% confidence intervals. As this is already clearly conveyed by the axis labels and panel titles, we do not consider it necessary to add a separate explanation in the figure caption. Notably, patients with both sarcopenia and high NLR had the highest risk of adverse outcomes. Compared with patients without sarcopenia and with low NLR, this group had a 2.48-fold increased risk of 90-day mortality (HR 2.48; 95% CI 1.40–4.40; *p* = 0.002) and a 1.81-fold increased risk of overall mortality (HR 1.81; 95% CI 1.37–2.38; *p* < 0.001), which were independent of clinical and donor-related covariates (Table 2, Supplementary Tables S1 and S2). Sarcopenia alone was also independently associated with both 90-day (HR 1.73, 95% CI 1.14–2.61; *p* = 0.009) and overall mortality (HR 1.60, 95% CI 1.30–1.98; *p* < 0.001), whereas high NLR alone was only marginally associated with 90-day mortality (HR 1.58, 95% CI 1.01–2.47; *p* = 0.045) and was not significantly associated with overall mortality (*p* = 0.134).

### 3.3. Subgroup Analysis

In the subgroup analysis stratified by age, sex, and MELD score, the combination of sarcopenia and high NLR was consistently associated with overall mortality across all subgroups, with the strongest association observed in women (HR 3.43, 95% CI 1.83–6.43; Table 3). The distribution of donor–recipient sex combinations is summarized in Supplementary Table S3.

**Figure 3:**
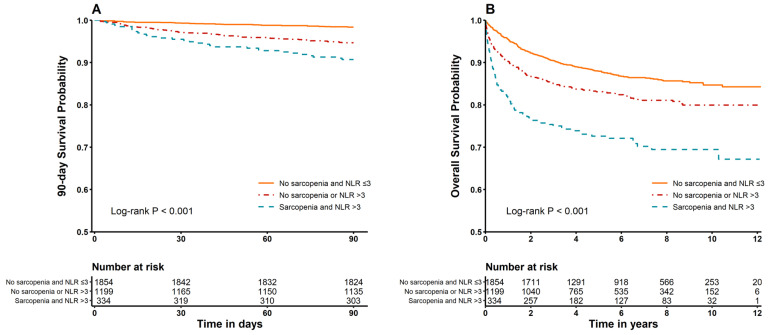
Kaplan–Meier curve of (**A**) 90-day mortality and (**B**) overall mortality according to NLR and sarcopenia status.

Kaplan–Meier survival analysis showed significantly lower survival in patients with both sarcopenia and high NLR compared to that in other groups (log-rank *p* < 0.001 for both 90-day and overall mortality) (Figure 3).

## 4. Discussion

The present study demonstrated that LT recipients with both sarcopenia and elevated NLR had the highest risk of short-term and long-term mortality. This association was consistently observed in the subgroup analysis stratified by age, sex, and MELD score, supporting the generalizability and clinical relevance of the findings. These results highlight the prognostic value of integrating inflammatory markers and muscle-mass assessment in pretransplant evaluation.

Our study findings, demonstrating the association between sarcopenia and systemic inflammation and the increased mortality associated with the co-occurrence of sarcopenia and systemic inflammation, are consistent with those of previous studies [20,21]. Notably, the co-occurrence of NLR > 3 and sarcopenia were associated with a higher mortality risk compared to the corresponding risk for each condition individually. In previous studies, sarcopenia [20,22] and systemic inflammation [14] were identified as significant factors affecting the adverse outcomes of patients undergoing LT. These findings complement previous studies on the prognostic significance of sarcopenia and inflammation in LT recipients. A recent systematic review and meta-analysis demonstrated that CT-assessed muscle mass was independently associated with both waitlist and post-transplant mortality, underscoring the value of objective muscle mass [4]. This view is further supported by expert consensus, which recognizes sarcopenia as a crucial factor to be considered in transplant evaluation and clinical decisions [23]. In addition, Leithead et al. identified NLR as a powerful predictor of mortality among LT candidates, independent of the MELD score [14]. However, few studies have investigated their combined prognostic effect, as in our analysis. Our results confirm and expand on these findings by demonstrating a synergistic impact of low muscle mass and systemic inflammation, even after accounting for established clinical and donor-related risk factors.

In another study involving 2470 patients with colorectal cancer, a comparison was made among patients classified based on the co-occurrence of NLR and sarcopenia; consistent with our findings, this previous study demonstrated that overall survival was worse in the group of patients with both elevated NLR and sarcopenia [24]. Furthermore, Halazun et al. demonstrated that preoperative NLR ≥ 5 has a negative impact on patients undergoing LT for hepatocellular carcinoma (HCC). They hypothesized that patients with a high NLR exhibit relative neutrophilia and lymphocytopenia, leading to an imbalance in the inflammatory cascade and the immune modulatory response against tumors. This imbalance was expected to impact tumor proliferation and metastasis [15].

This association was independent of the recipient’s characteristics, as reflected by MELD score, age, and sex. Our stratified analysis further supported this finding, demonstrating that the adverse prognostic impact of sarcopenia and systemic inflammation was consistently observed across all subgroups, and was most pronounced in women. Although the precise mechanisms underlying this sex-specific vulnerability remain to be fully elucidated, potential explanations include hormonal differences, variations in body composition, or sex-related immune responses that may increase susceptibility to the harmful effects of muscle depletion and inflammation [25]. These findings suggest that special attention should be paid to female recipients during pretransplant assessment and perioperative management.

In cancer patients, systemic inflammation is known to play a crucial role in tumor growth, progression, and metastasis [26]. In hepatocellular carcinoma, about 90% of the HCC burden is attributed to chronic inflammation due to viral hepatitis, excessive alcohol intake, or non-alcoholic steatohepatitis [27]. ESLD patients commonly present with systemic inflammation and immune system dysfunction. In advanced cirrhosis, bacterial translocation further activates the immune system, leading to an increase in levels of pro-inflammatory cytokines such as tumor necrosis factor-α (TNF-α), interleukin-1β (IL-1β), IL-6, and interferon-γ, and a decrease in levels of anti-inflammatory cytokines such as IL-10 and transforming growth factor-β [28].

Moreover, the current study revealed a negative correlation between the NLR and SMI. This relationship between systemic inflammation and sarcopenia has been well established in previous research. Elevated levels of inflammatory markers have a negative impact on skeletal muscle metabolism through direct or indirect mechanisms [29]. For instance, TNF-α and IL-6 were associated with muscle mass and muscle strength [30]. Muscle protein metabolism is regulated by a balance between muscle protein breakdown (MPB) and muscle protein synthesis (MPS); IL-6, as previously mentioned, reduces MPS [24]. TNF-α can also directly affect muscle protein degradation [31] and can contribute to the development of insulin resistance by reducing insulin-regulated glucose transporter levels and impairing the insulin signaling pathway [32,33].

In addition to the increase in pro-inflammatory cytokines, patients with ESLD often experience reduced physical activity and nutritional impairment, leading to an imbalance between MPS and MPB. This imbalance can result in the degradation of skeletal muscle mass. Additionally, insulin resistance plays a key role in sarcopenia [29]. Specifically, patients with ESLD are susceptible to developing sarcopenia, and there are numerous studies that report the association of sarcopenia with mortality and graft failure in patients undergoing LT [4,6,20].

Although body mass index (BMI) is a commonly used anthropometric measure, it does not accurately reflect skeletal muscle mass or distribution. In patients with ESLD, BMI may remain within the normal or elevated range due to factors such as fluid retention or adiposity, masking significant muscle depletion. This highlights the limitation of BMI in identifying sarcopenia and supports the use of imaging-derived skeletal muscle index (SMI) for a more accurate assessment. Our findings further underscore the clinical relevance of SMI by demonstrating its strong association with post-transplant mortality, even in patients who would not be considered underweight based on BMI alone.

Our study has some limitations. First, because this is a retrospective study, we cannot be certain that all possible confounding factors were considered. Although certain factors that influence the outcomes of LT were adjusted in the multivariable analysis, unpredicted confounding factors contributing to mortality may exist. However, to overcome the limitations of the retrospective design and influences of unpredicted factors, we validated our findings using the subgroup analysis including sex, age, and MELD score. Second, our data are derived from electronic medical records at a single medical center and are limited to patients of a single ethnicity. Therefore, further studies involving heterogenous groups of patients are necessary to validate the findings of our study.

## 5. Conclusions

In summary, patients with both sarcopenia and elevated NLR were at significantly higher risk of mortality after LT. As both markers are routinely measured and potentially modifiable, their combined assessment may enhance prognostic evaluation and may help guide targeted interventions.

## Figures and Tables

**Table 1:** Demographic and perioperative characteristics of recipients and donors in living donor liver transplantation.

	No Sarcopenia			Sarcopenia		
	NLR ≤ 3 (*n* = 1854)	NLR > 3 (*n* = 1058)	*p* Value	NLR ≤ 3 (*n* = 141)	NLR > 3 (*n* = 334)	*p* Value
Patients’ demographics						
Age, years	53 (48–58)	53 (47–58)	0.380	54 (48–59)	54 (47–60)	0.947
Male sex, *n* (%)	1390 (75.0)	699 (66.1)	<0.001	126 (89.4)	297 (88.9)	1.000
Body mass index, kg m^−2^	24.6 (22.7–26.7)	24.1 (22.1–26.5)	<0.001	21.1 (19.1–23.4)	20.8 (19.4–23.2)	0.992
MELD score	12 (9–16)	21 (13–33)	<0.001	17 (11–22)	26 (16–36)	<0.001
Diabetes, *n* (%)	394 (21.3)	271 (25.6)	0.008	43 (30.5)	84 (25.1)	0.276
Hypertension, *n* (%)	330 (17.8)	156 (14.7)	0.038	25 (17.7)	39 (11.7)	0.106
Beta blocker, *n* (%)	545 (29.4)	332 (a31.4)	0.280	41 (29.1)	109 (32.6)	0.513
Diuretics, *n* (%)	675 (36.4)	698 (66.0)	<0.001	81 (57.4)	238 (71.3)	0.005
Preoperative laboratory data						
Hemoglobin g dL^−1^	11.3 (9.7–13.0)	9.5 (8.4–11.0)	<0.001	9.4 (8.2–11.1)	8.9 (7.9–10.3)	0.002
Albumin, g dL^−1^	3.1 (2.7–3.6)	3.1 (2.7–3.5)	0.818	3.0 ± 0.6	3.1 ± 0.6	0.774
Sodium, mmol L^−1^	140 (137–141)	137 (133–140)	<0.001	137 (133–140)	135 (130–139)	0.003
Etiology of cirrhosis						
HBV, *n* (%)	1255 (67.7)	606 (57.3)	<0.001	73 (51.8)	154 (46.1)	0.304
HCV, *n* (%)	143 (7.7)	52 (4.9)	0.005	11 (7.8)	20 (6.0)	0.598
Alcoholic, *n* (%)	260 (14.0)	218 (20.6)	<0.001	34 (24.1)	113 (33.8)	0.047
Other disease, *n* (%)	234 (12.6)	197 (18.6)	< 0.001	26 (18.4)	59 (17.7)	0.944
Combined HCC, *n* (%)	1064 (57.4)	369 (34.9)	<0.001	49 (34.8)	87 (26.0)	0.071
Donor-related variables						
Donor Age, year	27 (22–33)	29 (23–39)	<0.001	28 (21–36)	32 (24–41)	0.001
Donor male sex, *n* (%)	1276 (68.8)	714 (67.5)	0.481	98 (69.5)	230 (68.9)	0.976
Donor body mass index, kg m^−2^	22.8 (20.9–24.7)	23.0 (20.9–25.0)	0.138	22.4 (20.2–24.8)	22.7 (20.8–24.9)	0.142
Graft-to-recipient weight ratio	1.05 (0.92–1.23)	1.19 (0.99–1.48)	<0.001	1.21 (1.01–1.41)	1.31 (1.06–1.91)	0.001
Deceased-donor graft	65 (3.5)	224 (21.2)	<0.001	17 (12.1)	104 (31.1)	<0.001
Intraoperative data						
Operation time, min	770 (691–865)	770 (679–881)	0.930	767 (682–856)	756 (655–875)	0.888
Cold ischemic time, min	82 (66–101)	86 (70–127)	<0.001	85 (66–101)	99 (78–193)	<0.001
Warm ischemic time, min	40 (33–50)	42 (35–52)	<0.001	40 (33–48)	43 (36–53)	0.013
Postreperfusion syndrome, *n* (%)	933 (50.3)	601 (56.8)	0.001	91 (64.5)	221 (66.2)	0.814
Massive transfusion, *n* (%)	540 (29.1)	625 (59.1)	<0.001	71 (50.4)	244 (73.1)	<0.001
Outcome						
90-day mortality	30 (1.6)	59 (5.6)	<0.001	5 (3.5)	31 (9.3)	0.049
Overall mortality	240 (12.9)	186 (17.6)	0.001	27 (19.1)	95 (28.4)	0.045

Values are expressed as the mean (± SD) or median (interquartile range) for continuous variables, and *n* (%) for categorical variables. MELD, Model for End-stage Liver Disease; NLR, neutrophil-to-lymphocyte ratio; HBV, hepatitis B virus; HCV, hepatitis C virus; HCC, hepatocellular carcinoma.

**Table 2:** Sarcopenia, neutrophil-to-lymphocyte ratio (NLR), and mortality after living donor liver transplantation.

	Univariate		Multivariate *	
	HR [95% CI]	*p* Value	HR [95% CI]	*p* Value
90-day mortality				
Sarcopenia				
No	1 [reference]		1 [reference]	
Yes	2.54 [1.72–3.74]	<0.001	1.73 [1.14–2.61]	0.009
NLR				
≤3	1 [reference]		1 [reference]	
>3	3.78 [2.56–5.59]	<0.001	1.58 [1.01–2.47]	0.045
Sarcopenia and NLR > 3				
Neither	1 [reference]		1 [reference]	
Both	5.97 [3.62–9.87]	<0.001	2.48 [1.40–4.40]	0.002
Overall mortality				
Sarcopenia				
No	1 [reference]		1 [reference]	
Yes	1.93 [1.58–2.37]	<0.001	1.60 [1.30–1.98]	<0.001
NLR				
≤3	1 [reference]		1 [reference]	
>3	1.63 [1.38–1.92]	<0.001	1.17 [0.95–1.42]	0.134
Sarcopenia and NLR > 3				
Neither	1 [reference]		1 [reference]	
Both	2.55 [2.02–3.24]	<0.001	1.81 [1.37–2.38]	<0.001

CI, confidence interval; HR, hazard ratio; NLR, neutrophil-to-lymphocyte ratio. Multivariable *, adjusted by backward regression: age, sex, Model for End-stage Liver Disease score, use of diuretics, hemoglobin, sodium, hepatitis B virus, hepatitis C virus, combined hepatocellular carcinoma, donor age, donor sex, graft-to-recipient weight ratio and deceased-donor graft.

**Table 3:** Sarcopenia, neutrophil to lymphocyte ratio (NLR), and mortality after living donor liver transplantation stratified by age, sex, and MELD score.

	Overall Mortality	
	HR [95% CI] ^a^	*p* Value
Age (years)		
<55	2.03 [1.35–3.04]	<0.001
≥55	1.59 [1.11–2.67]	0.011
Sex		
Male	1.54 [1.12–2.12]	0.008
Female	3.43 [1.83–6.43]	<0.001
MELD score		
<25	1.62 [1.09–2.40]	0.016
≥25	1.68 [1.03–2.73]	0.037

CI, confidence interval; HR, hazard ratio; MELD, Model for End-stage Liver Disease; NLR, neutrophil-to-lymphocyte ratio. HR ^a^, adjusted by backward regression: age, sex, Model for End-stage Liver Disease score, use of diuretics, hemoglobin, sodium, hepatitis B virus, hepatitis C virus, combined hepatocellular carcinoma, donor age, donor sex, graft-to-recipient weight ratio, and deceased-donor graft.

## Data Availability

The datasets used and/or analyzed during the current study are available from the corresponding author on reasonable request.

## Supplementary Materials

The following supporting information can be downloaded at https://www.mdpi.com/article/10.3390/jcm14165889/s1: Table S1: Multivariable Cox proportional analysis of 90-day mortality after liver transplantation; Table S2: Multivariable Cox proportional analysis of overall mortality after liver transplantation. Supplementary Table S3. Cross-tabulation of recipient and donor sex showing the distribution of same-sex and opposite-sex living donor liver transplantation.

## Abbreviations

The following abbreviations are used in this manuscript:

CT	computed tomography
DM	diabetes mellitus
ESLD	end-stage liver disease
HR	hazard ratio
HTN	hypertension
IL-1β	interleukin-1β
IQR	interquartile range
LC	liver cirrhosis
LT	liver transplantation
MELD	Model for End-stage Liver Disease
MPB	muscle protein breakdown
MPS	muscle protein synthesis
NLR	neutrophil-to-lymphocyte ratio
SD	standard deviation
SMA	skeletal muscle area
SMI	skeletal muscle index
TNF-α	tumor necrosis factor-α
