# Supplementary material for: Association Between Survival After Living Donor Liver Transplantation and Recipient Systemic Inflammation and Body Composition
Source: J Clin Med. 2025 Aug 20;14(16):5889. doi: 10.3390/jcm14165889 (PMC12387513; doi:10.3390/jcm14165889)
Supplement: Supplementary file 1 [file jcm-14-05889-s001.zip › jcm-3761449-supplementary.pdf]

Supplementary Table S1. **Multivariable Cox proportional analysis of 90-day mortality after liver transplantation**

	**Univariable**			**Multivariable**		
	**HR**	**95% CI**	***P* Value**	**HR**	**95% CI**	***P* Value**
No sarcopenia and NLR ≤ 3	1 [Reference]			1 [Reference]		
Sarcopenia ***or*** NLR >3	3.37	2.18-5.19	<0.001	1.50	0.92-2.44	0.101
Sarcopenia ***and*** NLR >3	5.97	3.62-9.87	<0.001	2.48	1.40-4.40	0.002
**Preoperative variables**						
Age	1.04	1.02-1.06	0.001	1.03	1.01-1.05	0.002
Male sex	0.55	0.38-0.79	0.001	0.59	0.4-0.87	0.007
Body mass index	0.99	0.94-1.04	0.560			
MELD score	1.07	1.06-1.08	<0.001	1.06	1.05-1.08	<0.001
Diabetes, *n* (%)	1.18	0.79-1.75	0.417			
Hypertension, *n* (%)	1.16	0.74-1.82	0.522			
Beta blocker	0.93	0.63-1.37	0.705			
Diuretics	2.85	1.91-4.24	<0.001	1.50	0.98-2.28	0.059
**Preoperative laboratory data**						
Hemoglobin g dL^{-1}	0.72	0.65-0.78	<0.001	0.92	0.82-1.02	0.097
Albumin, g dL^{-1}	0.90	0.67-1.2	0.474			
Sodium, mmol L^{-1}	0.99	0.96-1.02	0.495	1.03	1.00-1.05	0.078
**Etiology of cirrhosis**						
HBV, *n* (%)	0.44	0.31-0.62	<0.001	0.72	0.48-1.08	0.109
HCV, *n* (%)	1.93	1.13-3.31	0.017	1.73	0.96-3.11	0.068
Alcoholic, *n* (%)	1.29	0.84-1.96	0.241			
Combined HCC, *n* (%)	0.58	0.40-0.84	0.004	1.49	0.97-2.29	0.071
**Donor-related variables**						
Donor Age, yr	1.04	1.03-1.06	<0.001			
Donor Male sex, *n* (%)	0.71	0.5-1.02	0.062	0.69	0.48-1	0.047
Donor Body mass index, kg m^{-2}	1.01	0.96-1.07	0.730			
Graft-to-recipient weight ratio	1.31	1.21-1.42	<0.001			
Deceased-donor graft	4.62	3.22-6.63	<0.001			

CI, confidence interval; OR, odds ratio; NLR, neutrophil-to-lymphocyte ratio; MELD, Model for End-Stage Liver Disease.

Supplementary Table S2. **Multivariable Cox proportional analysis of overall mortality after liver transplantation**

	**Univariable**			**Multivariable**		
	**HR**	**95% CI**	***P* Value**	**HR**	**95% CI**	***P* Value**
No sarcopenia and NLR ≤ 3	1 [Reference]			1 [Reference]		
Sarcopenia ***or*** NLR >3	1.50	1.17-1.92	0.001	1.42	1.11-1.82	0.006
Sarcopenia ***and*** NLR >3	2.18	1.71-2.79	<0.001	1.56	1.19-2.04	0.001
**Preoperative variables**						
Age	1.03	1.02-1.05	<0.001	1.03	1.02-1.04	<0.001
Male sex	1.13	0.93-1.38	0.067			
Body mass index	0.98	0.95-1.00	0.067			
MELD score	1.03	1.02-1.04	<0.001	1.03	1.02-1.04	<0.001
Diabetes, *n* (%)	1.24	1.03-1.50	0.024			
Hypertension, *n* (%)	1.32	1.07-1.63	0.010			
Beta blocker	0.85	0.71-1.03	0.102	0.85	0.71-1.03	0.102
Diuretics	1.31	1.10-1.55	0.002			
**Preoperative laboratory data**						
Hemoglobin g dL^{-1}	0.90	0.86-0.93	<0.001	0.95	0.91-1.00	0.049
Albumin, g dL^{-1}	1.01	0.88-1.17	0.855			
Sodium, mmol L^{-1}	0.99	0.98-1.01	0.509			
**Etiology of cirrhosis**						
HBV, *n* (%)	0.77	0.65-0.91	0.003	0.81	0.66-0.99	0.040
HCV, *n* (%)	1.74	1.32-2.29	<0.001	1.43	1.05-1.95	0.022
Alcoholic, *n* (%)	1.01	0.81-1.26	0.915			
Combined HCC, *n* (%)	1.39	1.18-1.65	<0.001	2.16	1.76-2.64	<0.001
**Donor-related variables**						
Donor Age, yr	1.02	1.01-1.03	<0.001			
Donor Male sex, *n* (%)	0.93	0.78-1.11	0.431			
Donor Body mass index, kg m^{-2}	1.03	1.00-1.05	0.044			
Graft-to-recipient weight ratio	1.28	1.22-1.36	<0.001	1.37	1.25-1.50	<0.001
Deceased-donor graft	2.42	1.98-2.96	<0.001			

CI, confidence interval; OR, odds ratio; NLR, neutrophil-to-lymphocyte ratio; MELD, Model for End-Stage Liver Disease.

Supplementary Table S3. Cross-tabulation of recipient and donor sex showing the distribution of same-sex and opposite-sex LDLT

	Donor male	Donor female
Recipient male	320	517
Recipient female	693	1,696

LDLT, living donor liver transplantation.
